# High-Risk Fall Rehabilitation With Modified Otago Exercises and Medicinal Nano Gel Phonophoresis for Symptomatic Relief in Osteoarthritis of Knee

**DOI:** 10.7759/cureus.29308

**Published:** 2022-09-19

**Authors:** Medhavi V Joshi, Pratik Phansopkar

**Affiliations:** 1 Musculoskeletal Physiotherapy, Ravi Nair Physiotherapy College, Datta Meghe Institute of Medical Sciences, Wardha, IND

**Keywords:** case report, otago exercise, proprioception, osteoarthritis, knee pain

## Abstract

Proprioception is a major affection faced by individuals who undergo lower limb injuries. Especially in the elderly population, this places a major role in increasing the risk of fall. We are reporting a case of an elderly female having severe pain in her lower limb and difficulties in performing activities of daily living. Post clinical assessment, a functional diagnosis of osteoarthritic knee was made. The patient was managed with proprioception exercises (Otago exercises) and phonophoresis. These exercises were also beneficial in improving the apprehension of patient towards daily activities, walking, stair climbing, and fall risk. The technique of phonophoresis helped in alleviation of pain when given with a beneficial adjunct, like a medicinal gel.

## Introduction

Proprioception loss in elderly people creates a high risk of fall and a major problem under public health domain [1]. Physiological changes as a response of aging leading to the collateral damage to surrounding bone, soft tissue and ligaments contribute to the incident of fall. Since proprioception and lower limb strength is directly proportional to the balance, both static during standing and sitting and dynamic during transfers and walking, proprioception retraining and resistance training play an important role in the rehabilitation of elderly individuals [2].

Painful arthritic condition of knee is a major age-related change that damages the subchondral bone and synovial fluid, and creates an inflammatory response, a result of which there is persistent pain and reduced ability to perform functional instrumental everyday activity [3]. The growing sedentary lifestyle and unhealthy eating habits have also led to a higher increase in the rate of prevalence of obesity, with 46.51% in southern parts of Indian and lowest prevalence of 32.96% in eastern parts of the country, which is one of the major cause of arthritis painful knee condition.

The aim of physiotherapeutic rehabilitation in individuals with such risk of fall either secondary to osteoarthritis of knee, or primarily due to age-related changes and weakness, is symptomatic relief of pain and retraining for gaining lower limb strength and balance by using a modified supervised form of Otago exercise program [4].

We have utilized ultrasound in an electrotherapy modality for the purpose of symptomatic relief of pain [5]. Phonophoresis, an advanced form of ultrasound application that facilitates transdermal drug delivery has been administrated during the process. Diclofenac gel is most commonly used with the coupling medium for phonophoresis [6]. Since the patient was treated in a rural area of India, there was apprehension to use and unavailability of muscle relaxant gel. We have therefore combined ginger (*Zingiber*), a natural Ayurvedic medicinal herb with anti-inflammatory properties on topical application and oral intake, as an ingredient of the nano-gel phonophoresis [7,8]. The gel was formed by mixing aqua sonic gel and ginger gel in a ratio of 4:11 for therapeutic effect [9]. We thus report the effect of this medicinal nano gel phonophoresis in combination with Otago exercises as a single subject case report.

## Case presentation

A 62-year-old female retired professor presented with a history of persistent pain in right knee after standing for more than thirty minutes for last 4 months. The pain exaggerated after descending more than seven flights of stairs. There was no history of any trauma. The patient at the time had a BMI of 31.0 kg/m2 (grade I obese). On visual analogue scale, the intensity of pain on activity was seven. The pain had severely restricted the patient’s participation in social activities, which included managing events in her locality requiring standing for a long time.

Clinical assessment

Physical examination revealed no decrease in active knee range of motion bilaterally. On manual muscle testing, the grade for knee extensors and flexors was three. Hip flexors and extensors were both graded three plus. Hamstring tightness was present bilaterally. Tenderness of grade one on the medial aspect of the right knee with mild swelling was revealed by a figure of eight methods of measuring when compared bilaterally. Joint mobility was intact with the presence of crepitus. Observational gait analysis revealed more weight bearing on the left lower limb and an increased stance time on the same side with hiking of the right hip. With the above physical examination and keeping in mind the patient's endomorphic built and hectic lifestyle, we concluded osteoarthritis to be a provisional diagnosis. This was correlated with an X-ray which revealed grade three osteoarthritis according to Kallegren and Lawrence scale.

Outcome measures taken prior to the start of treatment were as follows: Visual Analogue Scale for pain, on activity, has intensity 7.3/10, WOMAC ( Western Ontario and McMaster Universities Osteoarthritis Index ) has a total score of 46%, and all the ranges were within normal values for hip knee and ankle. Findings for Star Excursion Balance Test are given in Table 1.

**Table 1:** Pre-treatment findings for Star Excursion Balance Test

Stance on Affected				Stance on Unaffected		
Direction	Value on Examination (v) in cm	Normalized to limb length (v/Limb length) in %	Average Distance	Value on Examination (v) in cm	Normalized to limb length (v/Limb length) in %	Average Distance
Anterior	63	80.76	72.75	65	83.33	74.83
Antero-Medial	49	62.82		51	65.38	
Medial	45	57.69		45	57.69	
Postero-Medial	57	73.07		59	75.64	
Posterior	62	79.48		63	80.76	
Postero- Lateral	60	76.92		63	80.76	
Lateral	61	78.20		62	79.48	
Antero-Lateral	57	73.07		59	75.64	

Investigations

**Figure 1:**
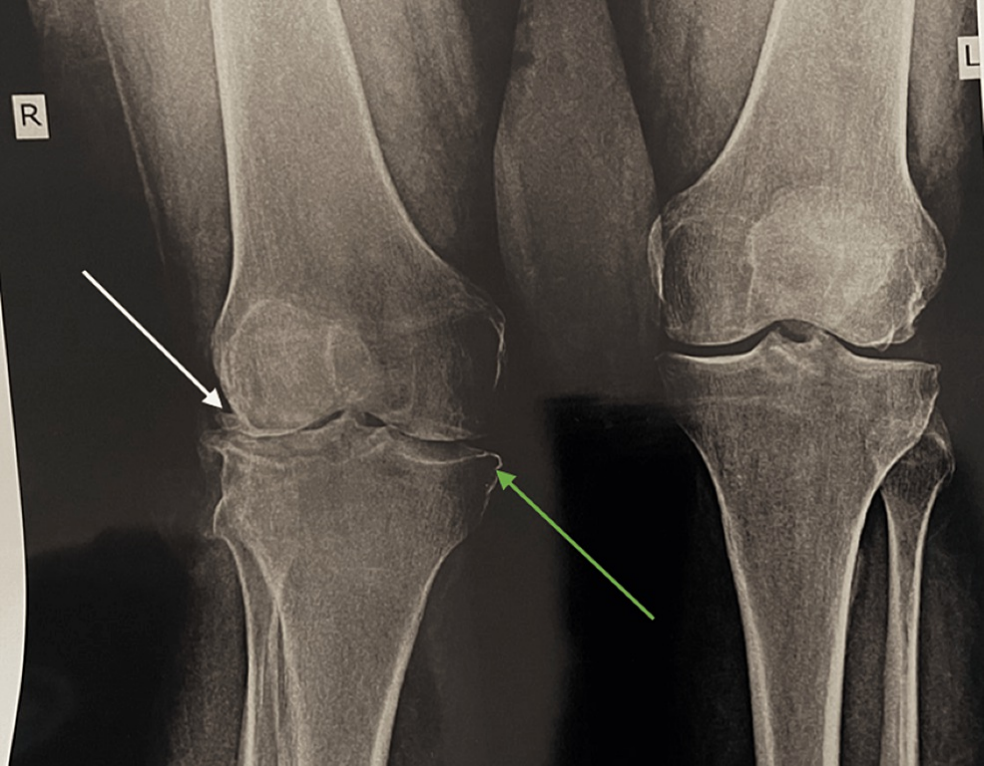
X-ray anteroposterior (AP) view It shows osteoarthritis of knee with joint space narrowing, sclerosis of bone, and multiple osteophyte formation representing grade three of knee osteoarthritis (OA) according to Kellgren- Lawrence scale.

Correlation of symptoms to the radiological investigations and the confirmation of the stage of knee osteoarthritis (KOA) with X-ray (Figure 1) was done.

Physiotherapy rehabilitation

As the pain was the major limiting factor of the functional activities, therefore a total of 10 sessions were taken in two weeks where both electrotherapeutic modality (Table 2) and an exercise program were given to the patient ( Table 3-4).

**Table 2:** Dosage of ultrasound: medicinal ginger gel was used for ultrasound phonophoresis

Variable	Duration/Intensity
Frequency	5 times a week for 2 weeks
Duration	40 minutes a week/2 weeks
Intensity	1.2 w/cm^2^
Mode	Continues

**Table 3:** Week one and two strengthening program

Exercise type	Week1	Week 2
Knee Extension	1.5 kg weight cuff 10 repetitions X 1 set	1.5 kg weight cuff 10 repetitions X 1 set
Knee Flexion		
Hip Abductors		
Ankle Plantar flexors	10 repetition X 2 sets with support	10 repetition X 2 sets without support
Ankle Dorsiflexors	10 repetition X 2 sets with support	10 repetition X 2 sets without support

**Table 4:** Balance re-training program

Exercise type	Week 1	Week 2
Knee bending	10 repetitions with support 2 sets	10 repetitions without support X 2 sets
Backward walking	10 steps, 4 times with support	10 repetitions, 4 times without support
Walking and turning around	Walk and turn around (figure of 8) twice	
Sideways walking	10 steps X 4 sets with yellow resistance band	
Tandem stance (heel-toe touch standing)	10 seconds X 2 sets with support	10 seconds X 2 sets with support
Tandem walk (heel-toe walk)	Walk 10 steps 4 times hold support	Walk 10 steps 4 times
One leg standing	10 seconds with support	10-second standing without support
Heel walking	10 steps 2 reps with support	10 steps 2 reps without support
Toe walk	10 steps 2 reps with support	10 steps 2 reps without support
Sit to stand	10 stands and two hands for support	10 repetitions without support.
Stair walking	4 flights ascend and descend thrice throughout day	8 flights ascend and descend thrice throughout day

**Figure 2:**
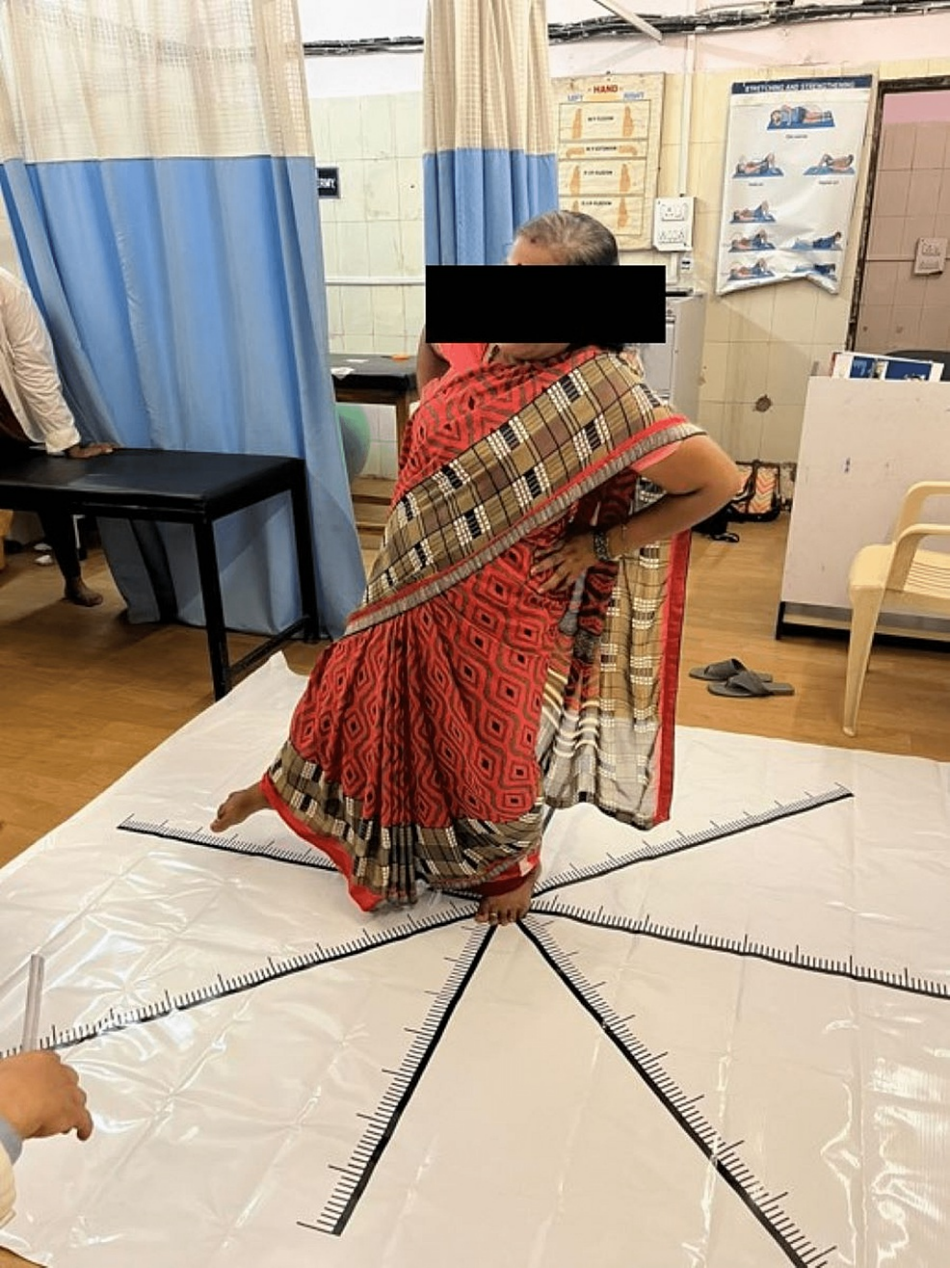
Star Excursion Balance Test Patient reaching out in lateral direction with stance on unaffected left extremity

Star Excursion Balance Test was also taken as an intervention where the patient performed clockwise reach-outs in all eight directions on the mat (Figure 2). Exercise was performed thrice daily only under supervision.

Follow-up and outcome measure

Post-treatment values for outcome measure are as follows: Visual Analogue Scale for pain, on activity, has intensity 2/10, WOMAC has a total score of 19%, and all the ranges were within normal values for hip knee and ankle. Findings for Star Excursion Balance test are given in Table 5.

**Table 5:** Post two-week treatment – outcome measure interpretation for Star Excursion Balance Test

	Stance on Affected			Stance on Unaffected		
DIRECTION	Value on Examination (v) in cm	Normalized to limb length (v/Limb length) in %		Value on Examination (v) in cm	Normalized to limb length (v/Limb length) in %	
Anterior	66	84.61538	79.967	66	84.61538	79.487
Antero-Medial	51	65.38462		51	65.38462	
Medial	49	62.82051		47	60.25641	
Postero-Medial	63	80.76923		63	80.76923	
Posterior	69	88.46154		69	88.46154	
Postero-Lateral	68	87.17949		68	87.17949	
Lateral	69	88.46154		68	87.17949	
Antero-Lateral	64	82.05128		64	82.05128	

## Discussion

We present a case of a woman who developed degenerative changes early in her life indicated by pain but neglected the same which lead to the development of a faster progression of the condition. The patient now presented with grade three of osteoarthritis in the early stages of her life. A previous history of knee ligamentous injury had led to altered joint loading in this patient.

Physiotherapy was implicated specifically to avoid fall risk as the patient had an initial apprehension towards even walking for 100m. A holistic approach that involved patient counselling, flexibility, strengthening and proprioception exercises was fabricated. A significant increase in the isotonic and isometric muscle strength for quadriceps, hamstrings, gastrocnemius and soleus is found to be effective in reducing the fall risk and improvement in functional activities. Gained strength also improved patient’s self-confidence [10].

Star Excursion Balance Test activity was initially difficult for the patient to perform in a rhythmic fashion. By the end of a week, movement in all eight directions could be performed without the affected leg touching the floor in between a single cycle could be performed, indicating an overall increase in strength. These findings are in accordance with other research where Star Excursion Balance Test, functional strength, and fall risk have been used as outcome measures [11,12]. 

## Conclusions

This case report provides a clinical framework for improving and managing motor functions in geriatric patients. This exercise program can be used in clinical settings for all patients under the same category as in the case report and for those who are rehabilitating post injuries, with modifications in the amount of weights used for strength training. Further studies are required to evaluate the beneficial effects of modified application of ultrasound and for their long-term follow-up.
